# Targeted next-generation sequencing identified novel mutations associated with hereditary anemias in Brazil

**DOI:** 10.1007/s00277-020-03986-8

**Published:** 2020-03-23

**Authors:** M. C. C. M. Svidnicki, G. K. Zanetta, A. Congrains-Castillo, F. F. Costa, S. T. O. Saad

**Affiliations:** Centro de Hematologia e Hemoterapia da Universidade Estadual de Campinas, (HEMOCENTRO/UNICAMP), Campinas, SP, Brazil. Rua Carlos Chagas, 480, Campinas, SP 13083-878 Brazil

**Keywords:** NGS, Hemolytic anemia, RBC membrane defect, Hereditary spherocytosis, Hereditary anemias

## Abstract

Hereditary anemias are a group of heterogeneous disorders including hemolytic anemias and hyporegenerative anemias, as congenital dyserythropoietic anemia (CDA). Causative mutations occur in a wide range of genes leading to deficiencies in red cell production, structure, or function. The genetic screening of the main genes is important for timely diagnosis, since routine laboratory tests fail in a percentage of the cases, appropriate treatment decisions, and genetic counseling purposes. A conventional gene-by-gene sequencing approach is expensive and highly time-consuming, due to the genetic complexity of these diseases. To overcome this problem, we customized a targeted sequencing panel covering 35 genes previously associated to red cell disorders. We analyzed 36 patients, and potentially pathogenic variants were identified in 26 cases (72%). Twenty variants were novel. Remarkably, mutations in the *SPTB* gene *(*β-spectrin*)* were found in 34.6% of the patients with hereditary spherocytosis (HS), suggesting that *SPTB* is a major HS gene in the Southeast of Brazil. We also identified two cases with dominant HS presenting null mutations in trans with α-^LELY^ in *SPTA1* gene. This is the first comprehensive genetic analysis for hereditary anemias in the Brazilian population, contributing to a better understanding of the genetic basis and phenotypic consequences of these rare conditions in our population.

**Electronic supplementary material:**

The online version of this article (10.1007/s00277-020-03986-8) contains supplementary material, which is available to authorized users.

## Introduction

Hereditary anemias (HA) are a genetically and phenotypically diverse group of disorders associated with mutations in more than 70 genes [1]. Genetic defects occurring in HA arise from a variety of different mutations that affect production of red blood cells in the bone marrow or cause red cell destruction due to defects in structural proteins of the cell, synthesis of the globin chains, or the expression of intracellular enzymes. Clinicians rely on morphologic/biochemical tests and clinical characteristics; however, these approaches fail to detect a percentage of the cases, and classification through these methods is not accurate [2]. Traditional laboratory workflow for HA diagnosis encompasses several lines of tests, adding cost and delay to the results. Thus, the genetic analysis would provide more informative clues for differential diagnosis, patient stratification, and genetic counseling.

Molecular diagnosis using routine sequencing approaches is impractical due to the large number and size of involved genes. In this context, targeted sequencing analysis using NGS appears as an advantageous tool since it enables simultaneous testing of several genes and has proven useful for accurate, rapid, and cost-effective diagnosis of several diseases, including hereditary anemias [3–7].

We developed a comprehensive NGS panel interrogating 35 genes related to red blood cell disorders, excluding hemoglobinopathies. The panel covered the coding regions, splice site junctions, and some regulatory regions, providing a high-throughput assay.

## Materials and methods

### Sample collection

A total of 36 samples of unrelated patients under clinical suspicion of hereditary anemia were included in this study. The available clinical characteristics along with osmotic resistance assay non-incubated or incubated with various salt solutions are presented in Supplementary Tables 1–3. All the patients with hemolytic anemia had splenomegaly and icterus. When available, first-degree relatives of the probands were also recruited for validation of the pathogenicity of potentially causative variants identified in the panel. Family members of patients with *SPTA1* null mutations (patients 24, 35, 27, and 18) were also evaluated; clinical and molecular findings were shown in Supplementary Figs. 1–4, respectively. All patients gave written informed consent to the study; all procedures were approved by the University Ethics Committee of Campinas University.

A reference sample (NA12878) from the Coriell Institute for Medical Research Repository (Coriell Institute, Camden, NJ, USA) was used for comparing sequencing data. This sample was evaluated in triplicate, along with the 36 patients selected for the study. The libraries of each of the replicates were individually prepared using HaloPlex amplification followed by NGS.

The suspected clinical diagnosis before genetic testing was obtained by blood cell assays as peripheral blood or bone marrow morphology, osmotic fragility assays, and enzyme activity assays.

### HaloPlex capture probe design

Our custom-made targeted NGS panel covers 35 genes known to be mutated in red blood cell diseases, including 13 genes causing membranopathies, 14 genes related to red blood cell enzymopathies, and 8 genes known to cause congenital dyserythropoietic anemia (CDA), as detailed in Table 1. All exons and the promoter regions of *ANK1* and *PKLR* genes were included. Coverage of the target regions was 99.63%.

**Table 1:** Genes included in our panel and related phenotypes

Phenotype	Genes included in the panel
Membrane defects	*SPTA1, SPTB, ANK1, EPB42, SLC4A1, EPB41, ADD2, GYPC/D, EPB72, PIEZO1, KCNN4, RHAG, ATP11C*
Enzyme defects	*G6PD, PKLR, GPI, HK1, PFKM/, PFKL, TPI1, PGK1, GSR, GSS, GCLC, UMPH1, AK1, ALDOA*
Congenital dyserythropoietic anemia	*PARP4, SEC23B, KLF1, CDAN1A, C15ORF41, KIF23, GATA1, ALAS2*

Targeted gene capture and library construction for NGS was performed using HaloPlex as described by the manufacturer (Agilent Technologies, Santa Clara, CA).

### Next-generation sequencing

Samples were equimolarly pooled and sequenced using 2 × 150 paired-end sequencing on the Illumina HiSeq 2500 instrument according to manufacturer’s protocol.

### Data analysis and filtering methods

Sequencing reads were aligned against reference genome (UCSC hg19), and variants were called and annotated using the SureCall software (v.3.5.1.46; Agilent Technologies) and CLC Genomic Workbench (Qiagen).

Variants that had metric values of read depth (coverage) less than 20, quality score less than 20, or resulted in synonymous amino acid changes were excluded.

Variants previously classified as pathogenic by databases such as ClinVar, HGMD, dbSNP, deleterious variants expected to produce truncated or abnormal protein, or splice site variants were considered as causatives.

Novel missense variants classified as pathogenic for at least three in silico algorithms such as: SIFT (http://sift.jcvi.org/), Mutation Taster (http://www.mutationtaster.org/), Mutation Assessor (https://omictools.com/mutationassessor-tool), and Polyphen-2 (http://genetics.bwh.harvard.edu/pph2/), among others, were also filtered as causatives.

The degree of evolutionary conservation of the encoded amino acid was estimated by Clustal Omega (https://www.ebi.ac.uk/Tools/msa/clustalo/). We also accessed the InterVar software [8] to predict pathogenicity of the variants according to ACMG Guidelines [9]; benign variants were excluded.

### Sanger sequencing

To validate the potentially pathogenic variants, bidirectional Sanger sequencing was performed using the BigDye Terminator Cycle Sequencing kit (Applied Biosystems, Life Technologies Corporation, Carlsbad, CA, USA) on a ABIPrism 3130xl Genetic Analyzer (Applied Biosystems, Foster City, CA, USA). Primer sequences are available upon request. Sequences were compared to reference sequences using CLC Sequence Viewer 7.8.1 (Qiagen, www.qiagenbioinformatics.com). Chromatograms were visualized with CHROMAS v.2.6.4 (www.thechnelysium.com.au).

## Results

Hereditary spherocytosis (HS) was presumed in 26 patients, hereditary elliptocytosis (HE) in 1 patient, enzymatic deficiency (ED) in 3 patients, and congenital dyserythropoietic anemia (CDA) in 4 patients. Additionally, 2 patients had inconclusive diagnosis due to absence of clinical and biochemical tests. Of the 26 patients with HS, three patients displayed a moderately severe phenotype, three patients displayed a moderate phenotype, and 14 presented mild HS (detailed clinical characteristics are showed in Supplementary Tables 1–3). Criteria for disease severity stratification have been previously defined [10, 11].

The comparison between the detected variants in our sample with exome sequencing databases of the reference sample NA12878 showed a sensitivity of 98.7% and specificity of 99.99% for the developed panel (Supplementary Table 4).

Potentially causative variants were identified in 26/36 (72%) of the subjects (Tables 2–4). In silico pathogenicity prediction by combined annotation-dependent depletion (CADD) score [12] was greater than 21.7 for all identified potentially pathogenic variants. The amino acid position conservation was 100% for all new missense variants. Deleterious variants with strong impact on the protein sequence including frameshift, nonsense, and splice site accounted for 64% (18/28) of the variants. Remarkably, 71% (20/28) had not been previously described and appear to be novel findings.

**Table 2:** Genetic variants, phenotype, and family history of 21 patients with membrane disorders

Patient	Previous diagnosis	Phenotype	Genes	Variants	Alleles	ID dbSNP/HGMD Accession	Annotation	InterVar/CADD score	α-LELY (Y/N)	Family history/evaluation
23	HS	*Moderately Severe*	*SPTB* (exon 3)	NM_000347:c.C361T (p.Q121*)	HT	New	Nonsense	Pathogenic/NA	Y	Mother with mild HS. Parental sample not available
28	HS	*Mild*	*SPTB* (exon 3)	NM_000347:c.T458A (p.I153N)	HT	New	Missense	Unc Sig/29.8	Y	Two children with HS. Parental sample not available
26	HS	*Mild*	*SPTB* (exon 3)	NM_000347:c.397_398del (p.M133fs)	HT	New	Frameshift deletion	Pathogenic/NA	N	NA/ Parental sample not available
13	HS	*NA*	*SPTB* (exon 7)	NM_000347:c.792delA (p.K264fs)	HT	New	Frameshift deletion	Pathogenic/NA	Y	Confirmed mutation in 2 sons with mild HS
7	HS	*Moderately Severe*	*SPTB* (exon 9)	NM_000347:c.1137_1138del (p.K379fs)	HT	New	Frameshift deletion	Pathogenic/NA	Y	No family history. Confirmed de novo mutation
21	HS	*Moderate*	*SPTB* (exon 13)	NM_000347:c.C2659T (p.Q887*)	HT	New	Nonsense	Pathogenic	Y	Son with HS. Parental sample not available
6	HS	*Mild*	*SPTB* (intron 19/20)	NM_000347:c.4267-1G > A	HT	New	Splice site	Unc Sig/NA	Y	Confirmed mutation in 2 daughters with mild HS
2	HS	*Mild*	*SPTB* (intron 19/20)	NM_000347:c.4266 + 1G > T	HT	New	Splice site	Unc Sig/NA	Y	Confirmed mutation in father with mild HS
5	HS	*Moderate*	*SPTB* (exon 20)	NM_000347:c.A4414T (p.K1472*)	HT	New	Nonsense	Pathogenic/NA	Y	Son with HS. Confirmed de novo mutation
35	HS	Mild	*SPTA1* (exon 13)	NM_003126:c.1629delT (p.I543fs)	HT	New	Frameshift deletion	Pathogenic/NA	Y (trans)	Confirmed frameshift mutation in daughter and brother with mild HS
24	HS	*Mild*	*SPTA1* (exon 17)	NM_003126:c.2239delA (p.T747fs)	HT	New	Frameshift deletion	Pathogenic/NA	Y (trans)	Confirmed frameshift mutation in daughter with iron deficit
18	HS	*Mild*	*SPTA1* (exon 29)	NM_003126:c.4110delT (p.L1370fs)	HT	New	Frameshift deletion	Pathogenic/NA	Y (cis)	Confirmed mutation in one affected brother with mild HS. Both with α-LELY
27	HS	*Mild*	*SPTA1* (exon 30)	NM_003126:c.C4201T (p.Q1401*)	HT	rs866240607/ NA	Nonsense	Pathogenic/NA	Y (cis)	Confirmed mutation and α-LELY in affected father with mild HS
46	HS	*Mild*	*ANK1* (exon 5)	NM_000037:c.G352A (p.A118T)	HT	New	Missense	Unc Sig/31	N	Confirmed mutation in affected son with mild HS
16	HS	*Moderate*	*ANK1* (exon 6)	NM_000037:c.C457T (p.Q153*)	HT	New	Nonsense	Pathogenic	Y	No Family history. Confirmed de novo mutation
30	HS	*Mild*	*ANK1* (exon 6)	NM_000037:c.564_565insCACG (p.A189fs)	HT	New	Frameshift insertion	Likely pathog/NA	Y	Mother with iron deficiency. Parental sample not available
1	HS	*Mild*	*ANK1* (exon 8)	NM_000037:c.T776C (p.L259P);	HT	New	Missense	Unc Sig/28.9	Y (HO)	Corfirmed mutation in mother with HS
8	HS	*Mild*	*ANK1* (intron 23/24)	NM_000037:c.2559-1G > A	HT	New	Splice site	Unc Sig/NA	Y	Confirmed mutation in two affected family members
33	HS	*Moderately severe*	*ANK1* (exon 33)	NM_000037:c.4057_4058insCC (p.Q1353fs)	HT	New	Frameshift insertion	Likely pathog/NA	N	Corfirmed mutation in father with HS
22	HS	*Mild*	*SLC4A1* (exon 13)	NM_000342:c.G1469A (p.R490H)	HT	CM994551/NA	Missense	Unc Sig/26.2	Y	Son with HS. Parental sample not available
31	HS	*Mild*	*SLC4A1* (exon 17)	NM_000342:c.C2278T (p.R760W)	HT	rs37391682/ CM951170	Missense	Unc Sig/25.6	N	Confirmed mutation in father with mild HS

*HS* hereditary spherocytosis, *HO* homozygosis, *HT* heterozygosis, *NA* not available, *Unc Sig* uncertain significance, *Y* yes, *N* no

**Table 3:** Genetic variants, phenotype and family history of 3 patients with enzyme deficiency

Patient	Diagnosis	Phenotype	Genes	Variants	Alleles	ID dbSNP/ HGMD Accession	Annotation	Intervar/CAAD score	α-LELY (Y/N)	Family evaluation
34	ED	Severe	PKLR (exon 8; 7)	NM_000298:c.1117_1118insCATGGGGGACA (p.M373fs); c.C993A (p.D331E)	HT	New; rs138476691 CM950949	Frameshift insertion; missense	Unc Sig/21.7	N	No family history. Parental sample not available
17	ED	Severe	PKLR (exon 10)	NM_000298:c.G1532A (p.G511E)	HO	CM1615474	Missense	Unc Sig/26.1	Y	No Family history. Parental sample not available
36	ED	NA	G6PD (exon 5)	NM_000402: c.C496T (p.R166C)	XL	CM1111163	Missense	Likely pathog 21.7	N	No Family history. Parental sample not available

*ED* enzymatic disease, *HT* heterozygosis, *HO* homozygosis, *XL* monoallelic X-linked inheritance

**Table 4:** Genetic variants, phenotype, and family history of 2 patients with congenital dyserythropoietic anemia (CDA)

Patient	Diagnosis	Bone marrow morphology	Genes	Variants	Alleles	ID dbSNP/HGMD Accession	Annotation	CAAD score	α-LELY (Y/N)	Family evaluation
25	CDA I	Internuclear bridges, multilobulated erythroblasts	*CDAN1* (exon 13; 17)	NM_138477: c.G2407T (p.G803*); C1939T (p.P647S)	HT	NA/NA rs760570690/NA	Nonsense, missense	Pathogenic/ Unc Sig/26.8	Y	No family history. Each parent has one recessive mutation
42	CDA I	Internuclear bridges, multilobulated erythroblasts	*CDAN1* (exon 19)	NM_138477:c.G2605A (p.V869M)	HO	rs370895637/ CM023036	Missense	Unc Sig/28.8	N	No family history. Parental sample not available

*HT* heterozygosis, *HO* homozygosis, *?* unknown type

Genetic variants were detected in *SPTB* gene (β-spectrin) in nine cases, in the *ANK1* gene (ankyrin) in six cases, in *SPTA1* gene (α-spectrin) in four cases, in *SLC4A1* gene (band 3) in two cases, in *PKLR* gene (pyruvate kinase) in two cases, in *G6PD* gene (glucose-6-phosphate-dehydrogenase) in one case, and in *CDAN1* gene (codanin) in two cases.

Positive family history was obtained in 15 patients. Samples of affected and/or non-affected family members were available for 17 out of 36 patients. As part of the validation of putative pathogenic variants, we used Sanger sequencing and the medical history of first-degree relatives of the probands to confirm association of the variant with the phenotype. De novo mutations were confirmed in three cases. All nine *SPTB* variants were in heterozygosis. Novel deleterious mutations expected to produce truncated β-spectrin were the most frequent (Table 2).

We identified two families (patient 24 and 35) with α^LELY^ in trans with a null SPTA1 allele segregating with mild HS phenotype. Some affected relatives of these two patients with the null allele and the absence of α-^LELY^ presented a very mild phenotype, characterized by the presence of few spherocytes in the blood smear, no alteration in osmotic fragility, and a slight increase in reticulocyte counts (Supplementary Figs. 1–2). Two other families (18 and 27) with null mutations in one *SPTA1* allele presented mild HS (Supplementary Figs. 3–4). The clinical characteristic of the affected patients is very similar to the relatives of family 35.

Of the three cases with enzymatic deficiency, variants in the pyruvate kinase gene (*PKLR*) were identified in two patients (17 and 34). In patient 17, the variant was present in homozygosis, and the condition is explained by inbreeding in the family. In patient 34, two different potentially pathogenic variants were identified in heterozygosis, one is a frameshift mutation not previously described. One asymptomatic male with extremely reduced activity of glucose-6-phosphate dehydrogenase had the *G6PD* p.R166C variant located in the X chromosome. This variant was described in 2012 [13] (Table 3).

Of the four patients previously diagnosed with congenital dyserythropoietic anemia (CDA), two (25 and 42) had variants in the *CDAN1* gene (Table 4), characteristic of CDA type I.

## Discussion

This study aimed to design a targeted NGS panel that could improve diagnosis and classification of patients with HA. We evaluated the performance of our panel in 36 patients with clinical suspicion of HA. Despite most of our patients had previous diagnosis based on clinical/biochemical tests, variants that explain the etiology of the hematological disorders were identified in 26 cases (72%), allowing genetic counseling and appropriate medical supervision for their family members carrying the deleterious variants.

A previous Brazilian study identified *ANK1* variants in only 10% of HS patients, suggesting that mutations in *ANK1* might not be as common in Brazil as described for the Northern European population, where *ANK1* mutations account for ~ 50% of HS cases [10, 14, 15]. On the other hand, heterozygous variants in the *SPTB* gene were present in approximately 25% of HS patients of European ancestry [15]. In our study, we identified SPTB variants in 34.6% and *ANK1* variants in 23.1% of the HS patients.

Despite patients with β-spectrin deficiency typically have a mild to moderately severe presentation of the disease and ankyrin defects being associated with a wider clinical severity ranging from mild to severe [16], the disease severity between β-spectrin- and ankyrin-deficient patients did not differ in our study; both were identified mostly in patients presenting mild HS (Supplementary Table 5).

Genuine nondominant HS where probands are compound heterozygous for α-spectrin genetic variants have rarely been described [16, 17]. Most of the reports identified null mutations associated with low-expression alleles, generally the α-^*LEPRA*^ (low-expression Prague) allele [17–20]. α ^LEPRA^ mutation (c.4339-99C > T) occurs in *SPTA1* gene in about 5% of Caucasians [17]. Interestingly α-^*LEPRA*^ was not identified in any individual of our study, corroborating previous unpublished results of our group.

The p.L1858V polymorphism in exon 40 of α-spectrin (*SPTA1*) is in linkage disequilibrium with a variant NC_000001.10:g.158587858G > A in intron 45, associated with partial skipping of exon 46. The allele containing both variants is known as α-^LELY^, a low-expression allele. The frequency of α-^LELY^ is around 23% in the general population (http://exac.broadinstitute.org/); however, we found a higher proportion in our sample (61%). These high frequencies of this variant were not observed in previous studies with non-affected Brazilian individuals and could reflect an anecdotal finding.

Null mutations in *SPTA1* gene were identified in four families with hereditary spherocytosis (18, 24, 27, and 35). The clinical characteristics between individuals with null mutations in only one allele were very similar (spherocytes in blood smear, high reticulocytes rate, and normal osmotic fragility) supporting that null mutations in *SPTA1* produce dominant mild HS. In two patients of these families (24 and 35), the α-^LELY^ were identified in trans with the null allele and appeared to aggravate the phenotype (higher reticulocytes, lower hemoglobin count, and increased osmotic fragility) in comparison to family members without the α-^LELY^ (Supplementary Figs. 1–2).

In hereditary elliptocytosis, it is well known that α-^LELY^ in trans to a missense mutation in alpha-spectrin enhances the severity of the disease [21–24]. In spherocytosis, however, one previous study concluded that α^LELY^ in trans to null HS allele of the *SPTA1* gene do not cause HS, due to the sufficient supply of spectrin to meet the needs of the membrane [19]. The divergence between this previous report and our results may be caused by differences in the genetic background or methodologies to classify the disease. Other studies have demonstrated that the clinical severity of HS varies considerably even within a single family, reflecting the genetic heterogeneity of the disorder.

The clinical diagnosis of patients with hereditary anemias is often difficult. Thus, tools for accurate genetic analysis become crucial, mainly in cases with an ambiguous phenotype. Despite increasing affordability and development of automated analyses of NGS, the identification of pathogenic variants in association with HA through sequencing panels remains challenging, due to the high price per sample and laborious data processing. We believe the budgetary limitations for broader use of NGS platforms would be solved with the gradual reduction of cost of this technology over time. Cost reduction will allow simultaneous evaluation of family members to establish the inheritance pattern, introducing testing of newborns with a family history of HA and confirmation of the pathogenetic role of the identified variants.

## Conclusion

The panel developed in this study proved to be effective for the detection of causative mutations in hereditary anemias and has also contributed to a better understanding of the genetic basis and phenotypic consequences of these rare conditions in the Brazilian population. Even though our study was mostly limited to cases with membrane disorders, the gene panel has the capacity to interrogate other causes of inherited anemias (e.g., enzyme disorders and CDA). The *SPTB* gene was the most frequent cause of HS in our sample, resulting in variable phenotypic severity. Twenty new variants were identified; most of them were null (nonsense or frameshift) mutations. The α-^LELY^ variant was identified in most cases and appeared to modulate the severity of HS when in trans to a null allele in *SPTA1* gene.

## Electronic supplementary material

ESM 1 (DOCX 12 kb)

ESM 2 (DOCX 29 kb)

Supplementary Figure 1 (PNG 525 kb)

High Resolution (TIF 326 kb)

Supplementary Figure 2 (PNG 438 kb)

High Resolution (TIF 236 kb)

Supplementary Figure 3 (PNG 369 kb)

High Resolution (TIF 193 kb)

Supplementary Figure 4 (PNG 456 kb)

High Resolution (TIF 237 kb)
