# Supplementary material for: Targeted next-generation sequencing identified novel mutations associated with hereditary anemias in Brazil
Source: Ann Hematol. 2020 Mar 23;99(5):955–62. doi: 10.1007/s00277-020-03986-8 (PMC7241966; doi:10.1007/s00277-020-03986-8)
Supplement: Supplementary file 1 — (DOCX 12 kb) [file 277_2020_3986_MOESM1_ESM.docx]

**Supplementary Figure Legends**

**Sup. Fig 1.** Family 35 clinical and genetic aspects. a) Family heredogram. The arrow indicates patient 35. Filled figures indicate the affected individuals. b) Electropherogram obtained by Sanger sequencing. The arrow indicates the variant spot. c) Clinical characteristics of the family.

**Sup. Fig 2.** Family 24 clinical and genetic aspects. a) Family heredogram. The arrow indicates patient 24. Filled figures indicate the affected individuals. b) Electropherogram obtained by Sanger sequencing. The arrow indicates the variant spot. c) Clinical characteristics of the family.

**Sup. Fig 3.** Family 27 clinical and genetic aspects. a) Family heredogram. The arrow indicates patient 27. Filled figures indicate the affected individuals. b) Electropherogram obtained by Sanger sequencing. The arrow indicates the variant spot. c) Clinical characteristics of the family.

**Sup. Fig 4.** Family 18 clinical and genetic aspects. a) Family heredogram. The arrow indicates patient 18. Filled figures indicate the affected individuals. b) Electropherogram obtained by Sanger sequencing. The arrow indicates the variant spot. c) Clinical characteristics of the family.
