# Supplementary material for: Targeted next-generation sequencing identified novel mutations associated with hereditary anemias in Brazil
Source: Ann Hematol. 2020 Mar 23;99(5):955–62. doi: 10.1007/s00277-020-03986-8 (PMC7241966; doi:10.1007/s00277-020-03986-8)
Supplement: Supplementary file 2 — (DOCX 29 kb) [file 277_2020_3986_MOESM2_ESM.docx]

**Supplementary Table 1**. Clinical characteristics of patients with Membrane Disorder

| **Patient (age)** | **Splenectomy** | **Transfusions** | **Hb (g/dL)** | **VCM (fL)** | **Hapt**  **mg/dL** | **Bilir** | **Ret** | **Osmotic Frag.** | **Blood smear** |
| --- | --- | --- | --- | --- | --- | --- | --- | --- | --- |
| 23 (34y) | Yes | Frequent | 14.5^+^ | 90.8^+^ | NA | 0.47^+^ | 2.3%^+^ | Increased | aniso, poikilo, sphero |
| 28 (91y) | Yes | No | 11.8 | 85 | NA | 11.7 | 1.10% | Increased | aniso, poikilo, sphero |
| 26 (51y) | No | No | 15 | 90.2 | 7.38 | 1.42 | 3.23% | Increased | aniso, poikilo, sphero, dacro |
| 13 (46y) | Yes | NA | 14.4^+^ | 94.5^+^ | 27.4^+^ | 4.6 | 1.97%^+^ | Increased | aniso, poikilo, sphero |
| 7 (38y) | Yes | Frequent | 17.1^+^ | 84.3^+^ | NA | 1.6 | 2.18%^+^ | Increased | aniso, sphero, acantho, |
| 21 (35y) | Yes | NA | 11 | 79.9 | 28.1 | NA | 7% | Increased | aniso, sphero, acantho, |
| 6 (80y) | Yes | No | 14.8^+^ | 91.3^+^ | NA | NA | 3.16%^+^ | NA | aniso, sphero |
| 2 (49y) | No | No | 14.7 | 92.2 | 7.38 | 2.8 | 7.4% | Increased | aniso, acantho, sphero |
| 5 (36y) | No | 2x | 9 | 76 |  | 1.3 | 10% | Increased (inc) | aniso, poikilo, sphero |
| 35 (72y) | No | 2x | 12.6 | 89.8 | 7.5 | 2.2 | 7.9% | Increased (inc) | aniso, poikilo, sphero |
| 24 (35y) | No | NA | 12.5 | 91.8 | 8.6 | 3.3 | 5.9% | Increased | aniso, poikilo, sphero |
| 18 (51y) | No | NA | 11.4 | 95.7 | 7.5 | 3.65 | 7.9% | Increased | aniso, poikilo, sphero |
| 27 (38y) | No | NA | 14.1 | 93.6 | 7.3 | 1.6 | 5.5% | Normal | aniso, poikilo, sphero |
| 46 (60y) | Yes | NA | 11.7 | 93.9 | 150^+^ | 3.1 | 7% | Increased | aniso, poikilo, sphero |
| 16 (10y) | No | No | 9.95 | 73.1 | NA | 3.9 | 9.8% | Increased | aniso, poikilo, sphero, dacro |
| 30 (45y) | No | No | 12.2 | 75.2 | 7.5 | 6.7 | 7.9% | Increased (inc) | aniso, poikilo, sphero, dacro |
| 1 (42y) | No | No | 11.4 | 96.3 | 51.5 | 1.5 | 7.8% | Increased | aniso, poikilo, sphero, dacro |
| 8 (25y) | Yes | NA | 17.4 | 86.8 | 100.5^+^ | 2.8 | 2.01% | Increased | spheroc, micro |
| 33 (31y) | Yes | NA | 10 | 77.5 | 103^+^ | 6.1 | 14% | Increased | Aniso, micro, hipercromia, |
| 22 (61y) | No | No | 12.2 | 101.7 | 6.6 | 1.7 | 5.3% | Increased | aniso, sphero, pinched cells |
| 31 (20y) | No | No | 11.7 | 81.3 | 9 | 1.6 | 8% | Increased (inc) | aniso, poikilo, ellipto, sphero |

+ Post splenectomy; Bilir.: Bilirrubin total; Hapt.: Haptoglobin; Ret.: Reticulocyte count; Inc.: Incubated with salt solutions.

Supplementary Table 2. Clinical characteristics of patients with Enzymatic Deficiency.

| **Patient (age)** | **Splenect.** | **Transfusions** | **Hb (g/dL)** | **VCM (fL)** | **Hapt.** | **Bilir.** | **Retic.** | **Osm. Frag.** | **Blood smear** |
| --- | --- | --- | --- | --- | --- | --- | --- | --- | --- |
| 17 (30y) | Yes | Yes | 7.9 | 74.7 | NA | NA | 3.2% | Normal | Aniso, left shifts |
| 34 (28y) | No | NA | 7.3 | 81.4 | 7.31 | 2.3 | 8.6% | Normal | Aniso, target cells, ellipto |
| 36 (NA) | No | NA | NA | NA | NA | NA | NA | Normal | NA |

+ Post splenectomy; Bilir.: Bilirrubin total; Hapt.: Haptoglobin; Ret.: Reticulocyte count; Inc.: Incubate

Supplementary Table 3. Clinical characteristics of patients with CDA.

| **Patient (age)** | **Hb (g/dL)** | **VCM (fL)** | **Hapt.** | **Bilir.** | **Retic.** | **Blood smear** | **Bone marrow morphology** |
| --- | --- | --- | --- | --- | --- | --- | --- |
| 25 (56) | 10.90 | 86 |  |  | 1.50% | NA | Pres. of internuclear bridges, multilobulated/ binucleated erythroblasts. |
| 42 (55) | 11.2 | 91.1 | 20.3 | 0.82 | 2.31% | aniso, poikilo, dotted basophils | Pres. of internuclear bridges, multilobulated/ binucleated erythroblasts. |

+ Post splenectomy; Bilir.: Bilirrubin total; Hapt.: Haptoglobin; Ret.: Reticulocyte count; Inc.: Incubate

Supplementary Table 4. Panel statistical parameters

| **Identified variants** | 79 |
| --- | --- |
| **Sensitivity = TP/(TP + FN)** | 79/ (79+1) = 98,7% |
| **Specificity = TN/(TN+FP)** | 13846*/ (13846* + 2) = 99,9% |
| **PPV = TP/(TP + FP)** | 79/ (79+2) = 0,975 |
| **PNV = TN/(TN + FN)** | 13846*/ (13846* + 1) =0,999 |

TP: True positives; TN: True negatives; PPV: Predictive positive value; PNV: Predictive negative value *Total of sequenced bases minus TP.

**Supplementary Table 5.** Clinical characteristics of patients with spherocytosis according to mutated genes.

| **Clinical characteristics** | ***ANK1***  **(*n* = 6)** | ***SPTB***  **(*n* = 9)** | **SPTA1**  **(n=4)** | **SLC4A1**  **(n=2)** | **Total**  **(*n* = 21)** |
| --- | --- | --- | --- | --- | --- |
| Males | 3 (50%) | 5 (55.6%) | 2 (50%) | 1 (50%) | 11 (50%) |
| Age; median (range) | 37 (10-60) | 48 (34-91) | 44 (35-72) | 40 (20-60) | 42 (10-91) |
| Family history | 4 (67%) | 6 (66.9%) | 4 (83.3%) | NA | 14 (55.6%) |
| **Disease severity** |  |  |  |  |  |
| Mild | 4 (67%) | 4 (44.4%) | 4 (100%) | 2 (100%) | 15 (71.4%) |
| Moderate | 1 (16.7%) | 2 (22.2%) | 0 (0%) | 0 (0%) | 3 (14.3%) |
| Moderately Severe | 1 (16.7%) | 2 (22.2%) | 0 (0%) | 0 (0%) | 3 (14.3%) |
| NA | 0 (0%) | 1 (11.1%) | 0 (0%) | 0 (0%) | 1 (4.8%) |

NA: Not available
