# Supplementary figures and images for: Targeted next-generation sequencing identified novel mutations associated with hereditary anemias in Brazil
Source: Ann Hematol. 2020 Mar 23;99(5):955–62. doi: 10.1007/s00277-020-03986-8 (PMC7241966; doi:10.1007/s00277-020-03986-8)

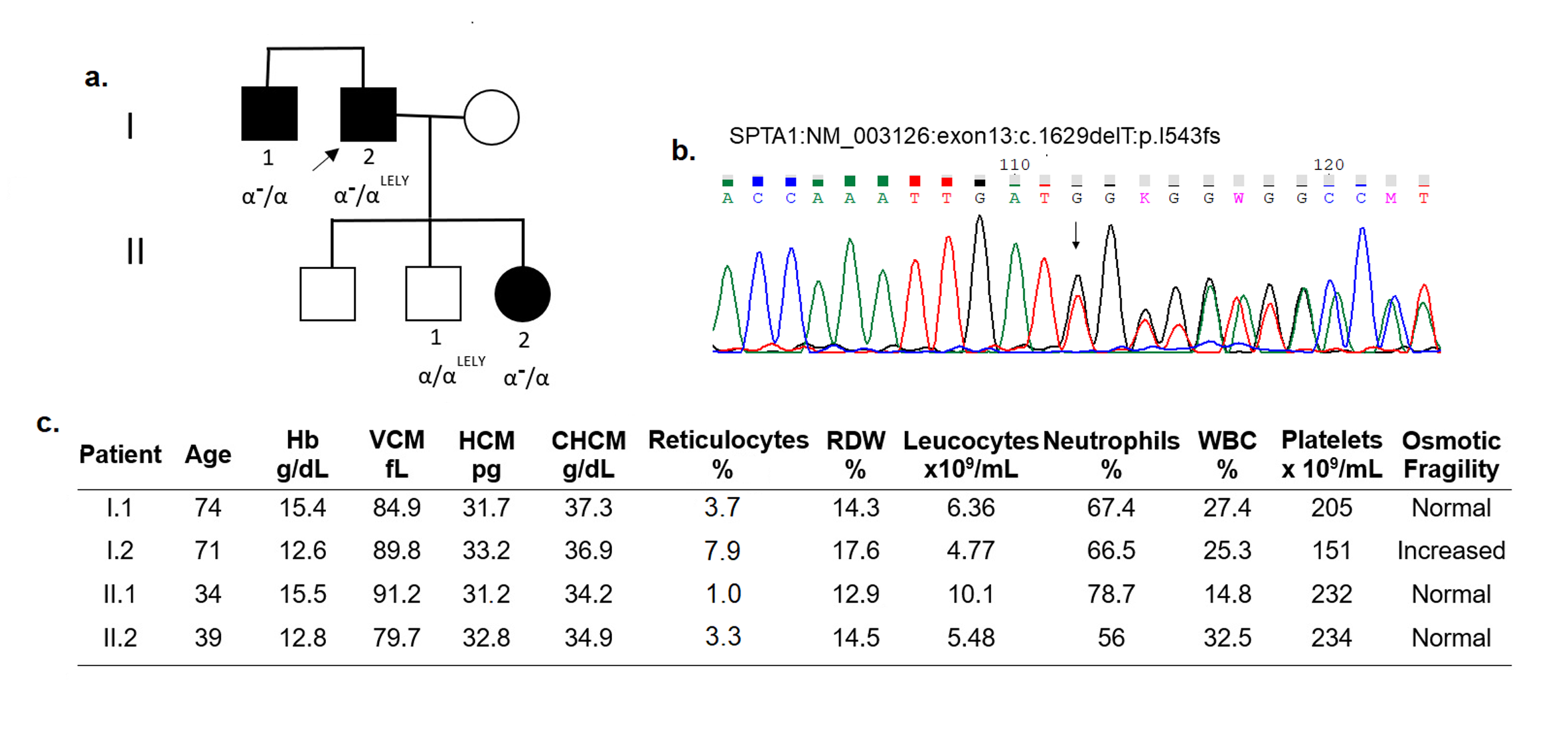

Supplement: Supplementary file 3 — (PNG 525 kb) [file 277_2020_3986_Fig1_ESM.png]

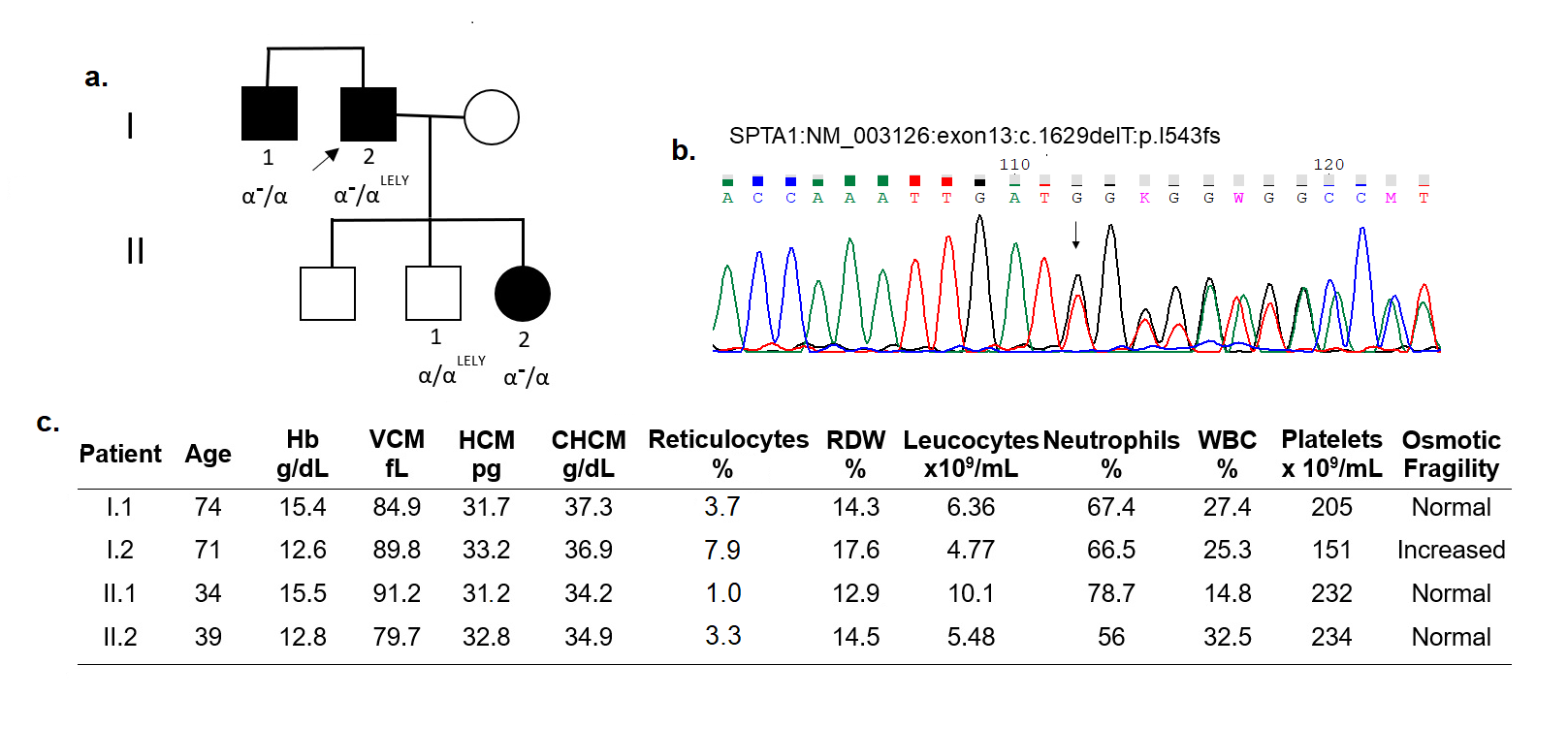

Supplement: Supplementary file 4 — High Resolution (TIF 326 kb) [file 277_2020_3986_MOESM3_ESM.tif]

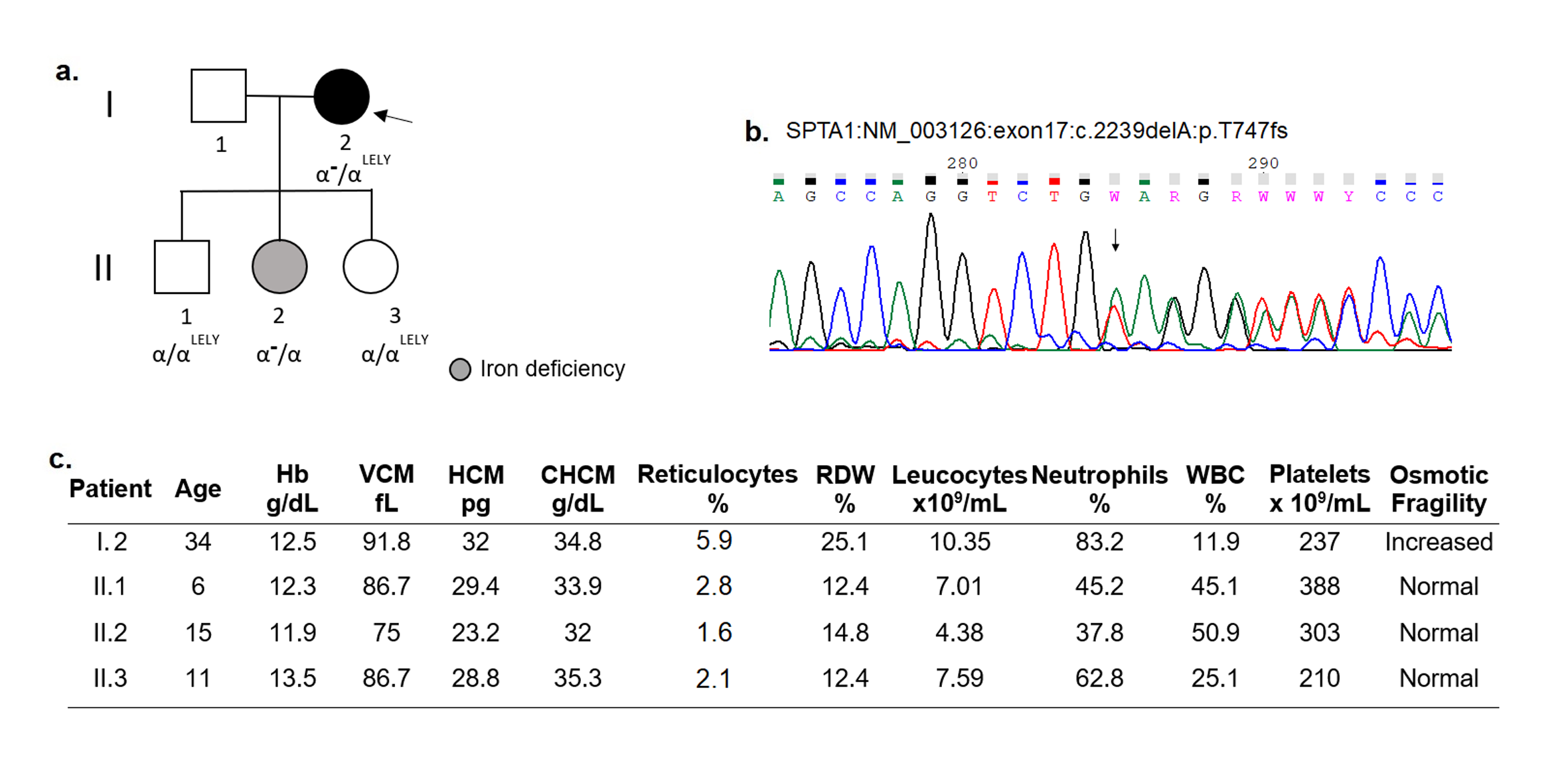

Supplement: Supplementary file 5 — (PNG 438 kb) [file 277_2020_3986_Fig2_ESM.png]

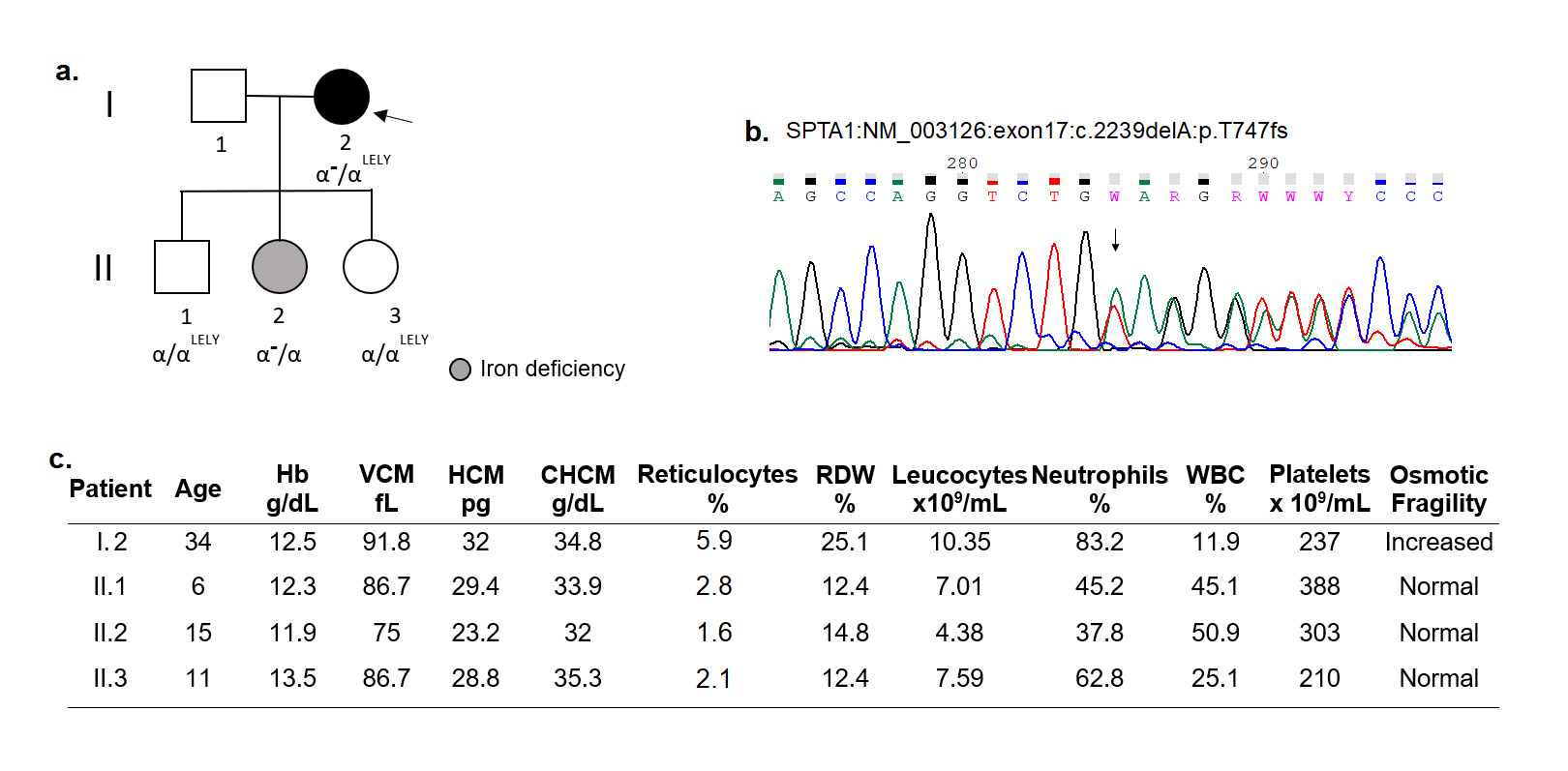

Supplement: Supplementary file 6 — High Resolution (TIF 236 kb) [file 277_2020_3986_MOESM4_ESM.tif]

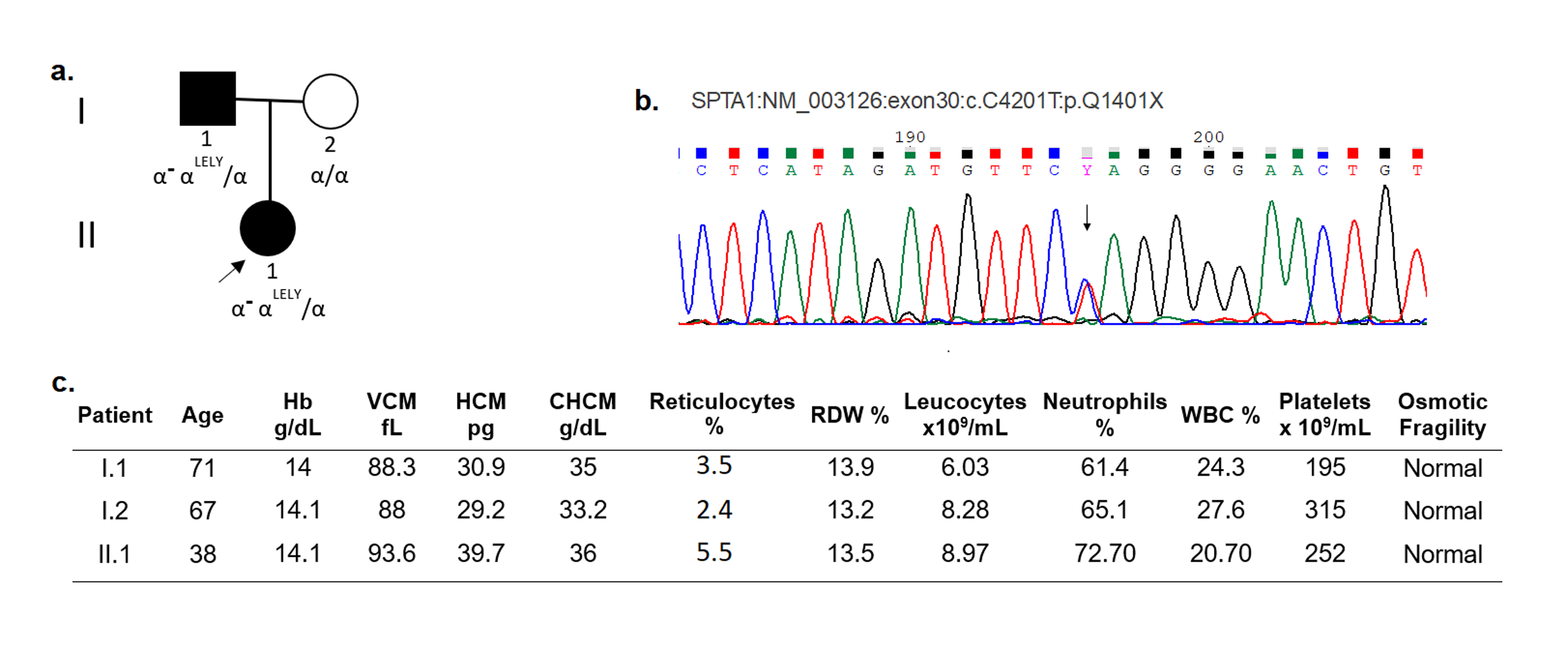

Supplement: Supplementary file 7 — (PNG 369 kb) [file 277_2020_3986_Fig3_ESM.png]

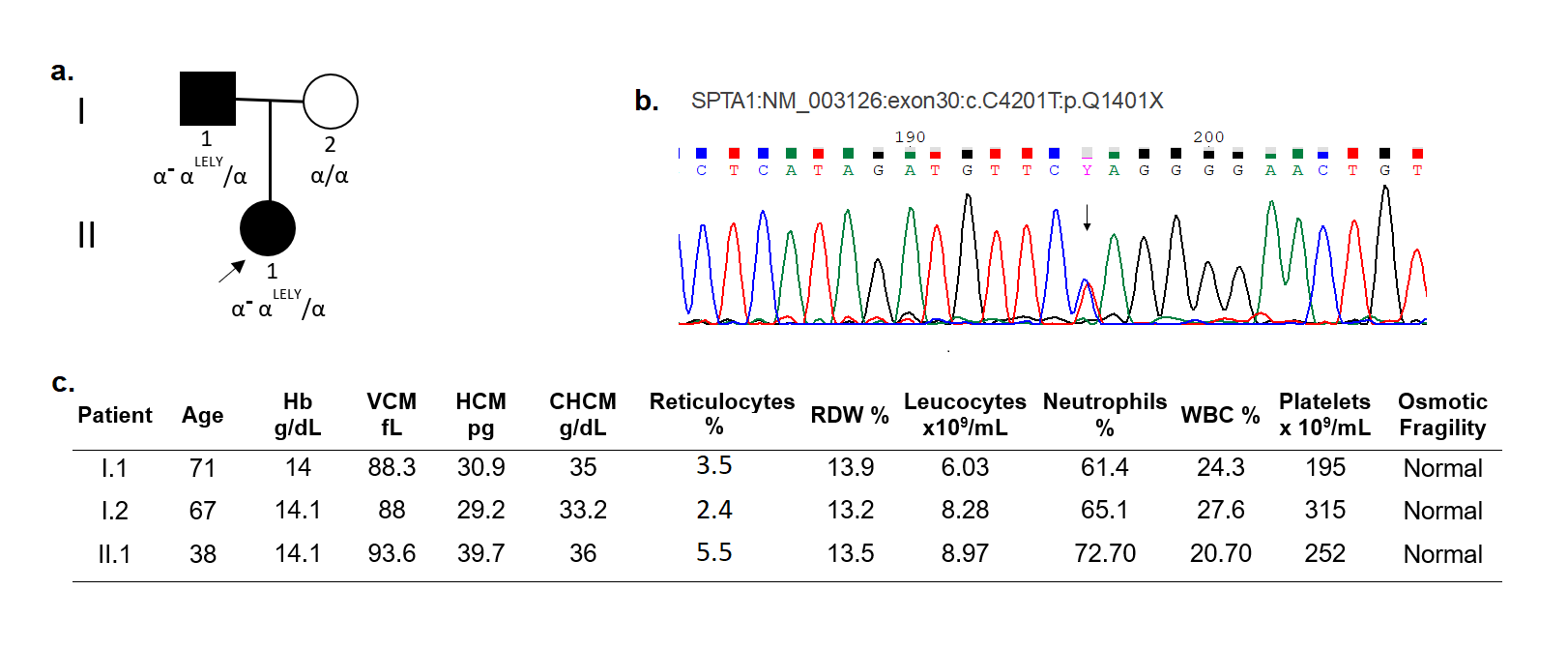

Supplement: Supplementary file 8 — High Resolution (TIF 193 kb) [file 277_2020_3986_MOESM5_ESM.tif]

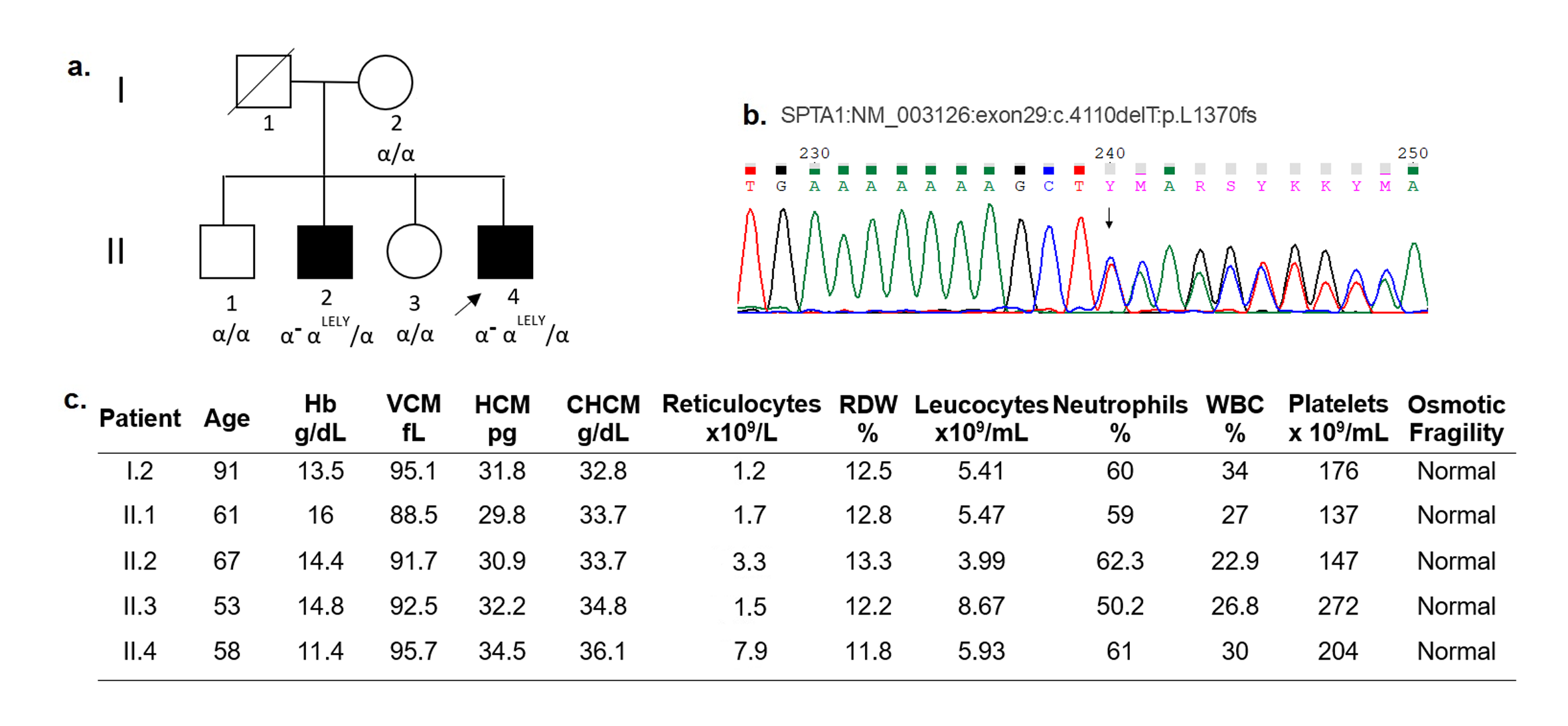

Supplement: Supplementary file 9 — (PNG 456 kb) [file 277_2020_3986_Fig4_ESM.png]

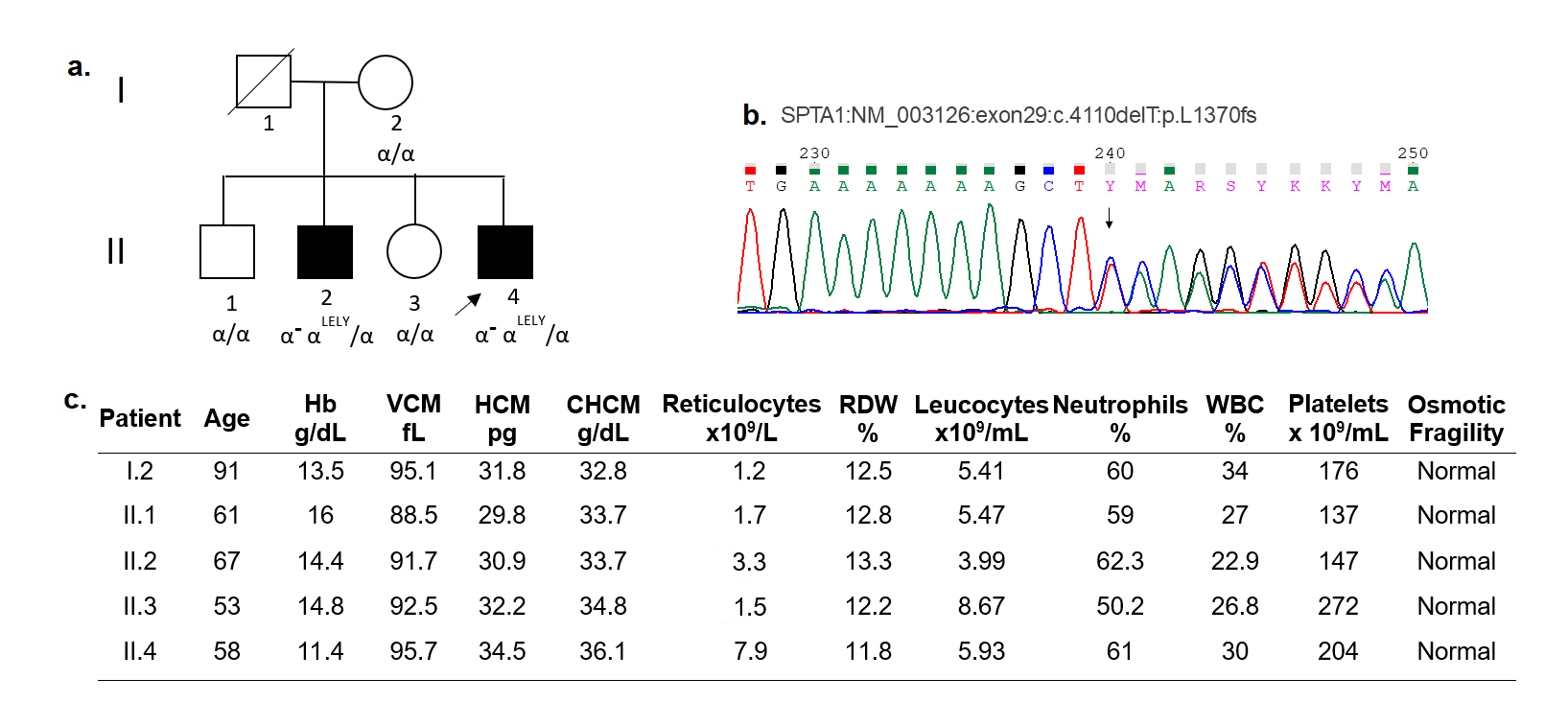

Supplement: Supplementary file 10 — High Resolution (TIF 237 kb) [file 277_2020_3986_MOESM6_ESM.tif]
